# Dental treatment for handicapped patients; sedation vs general anesthesia and update of dental treatment in patients with different diseases

**DOI:** 10.4317/medoral.19555

**Published:** 2013-10-13

**Authors:** José R. Corcuera-Flores, José M. Delgado-Muñoz, José C. Ruiz-Villandiego, Isabel Maura-Solivellas, Guillermo Machuca-Portillo

**Affiliations:** 1 Associated profesor in special care in dentistry, University of Sevilla, Spain; 2 Private practice in special care in dentistry, Quirón Hospital, San Sebastián, Spain; 3 Private practice in special care in dentistry, Nens Hospital, Barcelona Spain; 4 Chairman of special care in dentistry unit, University of Sevilla, Spain

## Abstract

Dental treatment on Handicapped Patients is often difficult because many people with a wide range of ages (from children to the elderly) with different pathologies that can affect the oral cavity and differ widely are included in this group. This situation creates some controversy, because according to pathology, each patient will be treated differently depending on collaboration, general health status, age or medication used to treat this pathologies. According to this situation we can opt for an outpatient treatment without any kind of previous medication, a treatment under conscious or deep sedation or a under general anesthesia treatment. With this systematic review is intended to help clarify in which cases patients should be treated under general anesthesia, sedation (conscious or deep) or outpatient clinic without any medication, as well as clarify what kind of treatments can be carried in private dental clinics and which should be carried out in a hospital. It will also discuss the most common diseases among this group of patients and the special care to be taken for their dental treatment.

** Key words:**Hospital dentistry, handicapped patient.

## Introduction

The lack of integration within the public health system is one of the most important problems of dental treatment for patients with special needs. Moreover, the peculiarity of decentralization of Public Health Services in Spain, makes public health patients with special needs coverage, very different within the same country (1).

This creates a lackof care that makes patients to keep looking for solutions to their dental problems in the field of private assistance (2).

We will present two examples of dental treatment integration in patients with special needs in a private hospital setting.

To evaluate the service provided to this type of patients, not only in Spain, a systematic review of the literature was performed using database PubMed-Medline using the keywords dentistry and handicapped patient hospital.

With the keywords used 151 articles were found, and then we proceeded to do a manual search to select the suitable items, select 22 to perform the review ( Table 1).

**Table 1:**
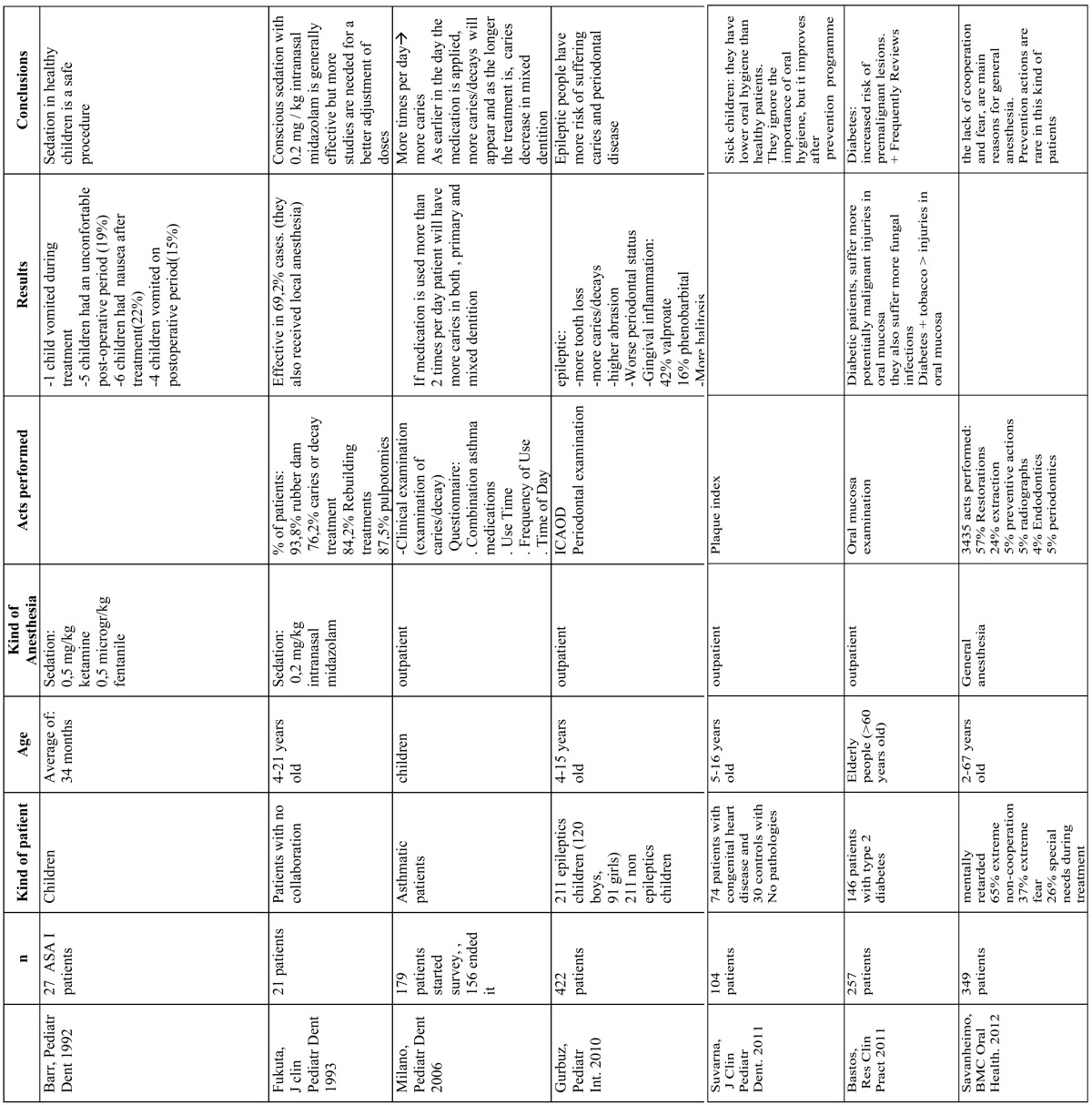
Most prominent research works in oral treatment of handicapped patients.

## Private hospital dental practice in adults

Private Hospital Dentistry, as a professional activity, only differs from the one made out in publics hospitals in the kind of patients who come or may come, and in the characteristics related to the way of work or the way in which assistance is provided.

On the other hand, indications, protocols, techniques, and ultimately, how patient is treated should be no different from a public or private one. But actually they have many distinguishing features: from how to capture the patient, the use of the cabinet and the operating room, the relationship with family and guardians, loyalty of patient and monitoring of treatment.

## Considerations

1. Public and private.

Understanding both , public and private assistance have the same purpose, oral and dental health. It is clear that the only difference between both assistance services is that in the public assistance, patient does not pay directly and he does it in the private one, with differences that it entails at all levels (from the management, training, technology, treatment options, human resources).

2. Hospital option.

Dental Clinic understood, as usual, (private, not big clinic with only 1 or 2 dentists, open to everybody) has its essence in business logic: It is private medicine that is practiced as close as possible to patients.

Hospital Dentistry is often only associated with the use of the operating room as a procedure room. It is a very simplistic and logical, derived from the tradition in the practice of dentistry.

Reality is very different.

Hospital Dentistry can not be only a pure Dentistry activity, because it allows dentistry to expand to large spaces of Stomatology and Oral Surgery. It could open dentistry to a prominent place in medicine, resulting from interaction with other hospital acts and medical specialities. It Can include all “Dental Clinics” features and may offer more.

Moreover, hospitals are those who are reluctant to provide space for dentistry because of its characteristics, that make dentistry not very related to other medical specialities.

Privates hospitals are usually interested in renting rotating spaces to different specialists better than one that also does not generate other benefits added to hospital.

Private hospital dental model.

After consider options where we can work, and once decided by Hospital dentistry, it is appropriate that we take into account several factors that will determine the success of this model that includes two types of patients.

• Patients without underlying systemic diseases.

• Handicapped Patients or patients with systemic diseases.

a) Patients without underlying systemic diseases:

Dentistry practiced in a Hospital competes with multitude of common dental clinics full of arguments for them: the next customer location, integration into the commercial life of the neighborhoods etc.

Then there is the assumed tradition by patients in concept of Dentist (something more aesthetic and associated with repairing teeth) and in concept of Hospital (associated with diseases).

Hospitals are in most cases associated to sophisticated treatments, technological advances and specialization these are important arguments that we should offer to conquer a reference position, position that will give us the famous “word of mouth” (patients talking with other people about our services) and can be reinforced with simple advertising campaigns.

Another essential aspect for the proper positioning within the Hospital of our dental service is to be able to choose the location close to fields related to our workspace: ENT, Dermatology, Plastic Surgery, Pediatrics, Psychiatry/Psychology (3).

Looking to our hospital, like any other specialty, you need to be more than a tenant: you should generate traffic of patients that can consume in other departments (Analytical, Rx, interdepartmental, operating rooms). This, which could be required from Hospital direction, comes up by activity that generates working in a hospital enviroment. In fact, from the beginning, we will find care demands not only in teeth areas and also we could find complex patients who have not been successful treated in their regular dentists, and who need dental attention in other environment (4).

b) Handicapped Patients.

Probably the most important differential area compared to Dental Clinics.

The special care in dentistry.

Those people who for physical or mental characteristics can only be treated in hospital environment, either for monitoring and controlling risks or because the only way to access to their mouth, is using general anesthesia or other form of sedation.

It’s easy, even today, associate this operation to the public health service but since transfers were initiated to differents Spanish health services, we find many contradictions and discrimination for being resident in one or another part of the country (5).

Additional cost that Dental performance in these kind of patients means, does, in many cases, impossible to treat them under the private health system when they need General Anesthesia, and they only have access to dental health through mutilation (extractions multiple) or the bounty of the Administration.

At this point is very important that hospital direction could get involved to promote patients referral with some agreements that can range from the “total” (completely covered dental care for patients with disabilities) to “partial”promoting access to the hospital (General Anes-thesia and Operating Room) and giving only economic coverage overall performance (extractions and surgery).

This simple formula, a combination of public and private health services, allows to do more just and equitable dental practice: handicapped patient can be treated at all levels at the same price as everyone else in the field of dentistry, and has the same right to Oral Health than any other person without pathologies because the state could provide additional medical facilities (hospital, operating room and anesthesia) for dental care.

Probably this health service model could be the final format that can be applied by the state because of economic rationality and social justice .

But from the point of view of independent dental professional, it is possible that this patient loads is not enough itself to maintain Dental Service.

Again is necessary to apply business judgment in this kind of professional dedication to find profits and for Monitoring evolution and loyalty of patients, promote oral health in those centers which care of disabled patients, collaborate in monitoring and maintaining hygiene and also promote a familiar motivation.

These actions, carried out systematically and notarized, have enabled successful experiences in creating Dentistry Services at Private Hospitals. Being also an obvious fact that morbidity and dependence in chronic patients in our society have increased. The incidence of cerebral palsy with a neonatal origin have been maintained and even decreased but dependencies related to age and senile dementias have gradually increased (6).

All of them have teeth as positive / negative factor in systemic health and they need to be treated.

Guidelines for special attention to handicapped patients in a private hospital.

Some private hospitals have technical resources to perform under sedation or general anesthesia oral treatments in both, normal patients and in handicap pedpatients. But not all of them have a Dental Service that could provide dental care in different specialties (as General Dentistry, Preventive Dentistry, Orthodontics, Speech, Maxillofacial Surgery, Special care in Dentistry) integrated within the hospital structure and related with other medical areas that can provide a comprehensive, multidisciplinary solution to health problems with joined protocols appropriate to patients needs.

Handicapped Patients in Pediatric Dentistry

Pediatric patients with special needs include a large number of patients with many different diseases that can be classified into 4 groups:

- Group 1 would be medically compromised patients.

- Group 2 include patients with motor deficits to a greater or lesser degree.

- Group 3 would consist of those with some sensory deficit (DEAF, BLIND people).

- Group 4 would bring together all patients with pathologies with some degree of intellectual disability.

Most of these patients, due to wide range of general pathology that may present, requires specially trained professionals, and individualized treatment plans that cover dental treatment needs.

In all cases we should always perform a complete clinical history, we should also request medical reports and study the underlying pathology. We should also know the medical treatments that are being performed as the same time of dental attention.

On the other hand, we must perform a dental history aimed to obtain an individual and objective assessment that will show us the most appropriate oral therapeutic needs in each case and will help us to decide what would be the best way to perform dental attention in clinic or under sedation or general anesthesia (7-9).

All of this makes necessary an interrelation and intercommunication with other medical and dental specialties, developing integrated protocols in many cases.

If all these actions could be coordinated from a hospital dental service that can organize referrals to other services (not only related to mouth) integrated in the same hospital, it will make everything easier, because not only number of movements of patient get reduced, because visits and tests could be jointly programmed, but, thanks to the computerization of services, it faci-litates access from any specialty to all tests that have been done to that patient.

## Clinical dental treatment in handicapped and madically compromised patients

Within this diverse spectrum of individual needs in dental treatment of handicapped patients, we must consider on one hand the underlying disorder which will mark the most appropriate treatment decisions, the different medical specialists who must intervene to schedule referrals and which protocols must be followed in each case.

In case of medically compromised pediatric patients, we should always perform a complete clinical record and we should also ask their doctors for a full report of their illness, treatment and an update prognosis. When patients come for the first time to our clinic, we always ask them from 5 years of age onwards, about an orthopantomography which can be performed the day before our appointment. Oral treatment may be performed in normal clinical following special protocols for each specific type of pathology and in some cases it will require coordination with others specialists who could directly control overall patient´s illness.

Patients from this group, usually attend a Hospital Dental Service because they feel safer getting dental care in this environment.

Pathologies that medically compromised patients could present can be very large and diverse and always require an overall assessment of patient status, to design individually dental treatment plan which suits better, each case needs.

Diabetic patients have no specific oral manifestations. For dental practice we must always control diabetes. Poorly controlled patients should be referred to specialist some days before dental treatment to control blood sugar levels.

It is recommended to perform dental treatment within two hours after the insulin injection and we should not modificate patient´s usual breakfast, not change medication schedules and especially food intake, both, before and after treatment.

Local anesthesia can be performed normally. We should remember that in these patients healing is slower and therefore, if extractions are performed, we recommend coverage with high spectrum antibiotics (10,11).

The most common complication in this kind of patients is hypoglycemia, which usually can be solved with administration of orally quick absorption carbohydrates (or parenterally), but we can avoid it easily if we adapt schedules appointments to patient´s intake.

Another group of patients are those who suffer from cardiac diseases. In these cases, patients who come more often are those with congenital cardiac abnormalities and those with functional murmurs. In first group we must always request specialist report (where should appear pathology´s current situation) , because is specialist who makes pathology following. We should always apply prophylaxis recommendation for bacterial endocarditis prevention in cases where it is indicated (12,13). In the second group, treatment does not require any special preparation.

In pediatric patients, breathing problems we see more frequently are those associated with bronchial asthma. Most common oral abnormalities in this kind of patients are an increased predisposition to tooth decay, associated with prolonged use of steroids and other drugs in suspension form for inhalation and dispensed daily (14).

It is recommended to perform Dental treatment in these patients in periods in which child is asymptomatic, appointments should be done at morning hours because we can monitor better patient´s situation, anesthetic with vasoconstrictor can be used, always performing aspiration during injection and should be avoided anesthetics with sulphites.

After inhaler use it is recommended to these kind of patients to always rinse their mouth with water, to avoid decay risk (14).

In epileptic patients without intellectual disabilities who take regular medication, are well controlled and who usually have no crisis, dental treatment can be normally performed at clinic. It is recommended to treat these kind of patient within 2 hours after they have consumed their medication.

In these patients we should avoid triggers factors for epilepsy, such as stress and anxiety. If during treatment, suddenly, a crisis appears, we should immediately remove all tools and materials from mouth, put patient in a supineposition, wemust tilt patients head sideways, and we should avoid mouth closing for avoid tongue biting (15).

For patients with mild motor deficits involving only arms and legs and not accompanied by intellectual disabilities, there won´t be any problem for normal dental treatment. In cases which shortfall affects upper limbs it is important to involve their family in proper daily dental hygiene.

Patients with sensory deficits, which more often come to clinic are those with impaired hearing ability or deaf patients, who in earlyonset cases it will probably be associated with speech deficits. In these cases, patient communication is the most important problem, which will make treatment exclusive and personalized: You need a substantial visual communication and specialized collaboration in sign language by their family or by a professional (16).

## Dental tratment of handicapped and medically compromised patients in an operating room

Within resources that can be provided in a hospital, one of the most important and requested by handicapped patients is dental treatment under general anesthesia or sedation (17,18).

Within this group of patients we can find medically compromised patients (congenital cardiac abnormalities, blood dyscrasias, allergic reactions to local anesthetics,. uncontrollable epilepsy, etc.) On the other hand, we have all the patients with motor deficits that don´t allow proper treatment in clinics and all patients who have a mild or severe intellectual disability, whose condition or treatment inhibit a dental treatment in clinics (8,16).

This group of patients (intellectual disabled) generally presents big problems, with many different oral pathologies, because they themselves are not able to seek medical care and disability also involves a failure to perform a proper daily oral hygiene and proper maintenance and it is also the group of patients which generates a greater request for hospital treatment under general anesthesia or sedation.

Inside this group we can find genetic intellectual disabilities such as Down syndrome, fragile X syndrome, Angelman syndrome etc. Others would be patients with severe neuromuscular disorders such as cerebral palsy and spina bifida we can also include in this group patients with autism spectrum disorders, affected by Asperger Syndrome and Rett Syndrome.

To assess these patients, we must make a general record of underlying disease with complete laboratory blood analysis and electrocardiogram , and dental history (orthopantomography etc.). With these data we can make an inquiry with the anesthesiologist to determine the health risk presented by the patient.

For this purpose the ASA group classification is used, which is a 6-degree scale created by the American Society of Anesthesiologists that relates the degree of surgical risk in the patient with his main pathology and with that, he will determine the type of anesthetic technique, general anesthesia or sedation, to perform dental treatment plan with the most appropriate option (17-19).

To achieve sedation treatments patients can be only candidates if they are included in both ASA groups I and II ( Table 2).

**Table 2:**
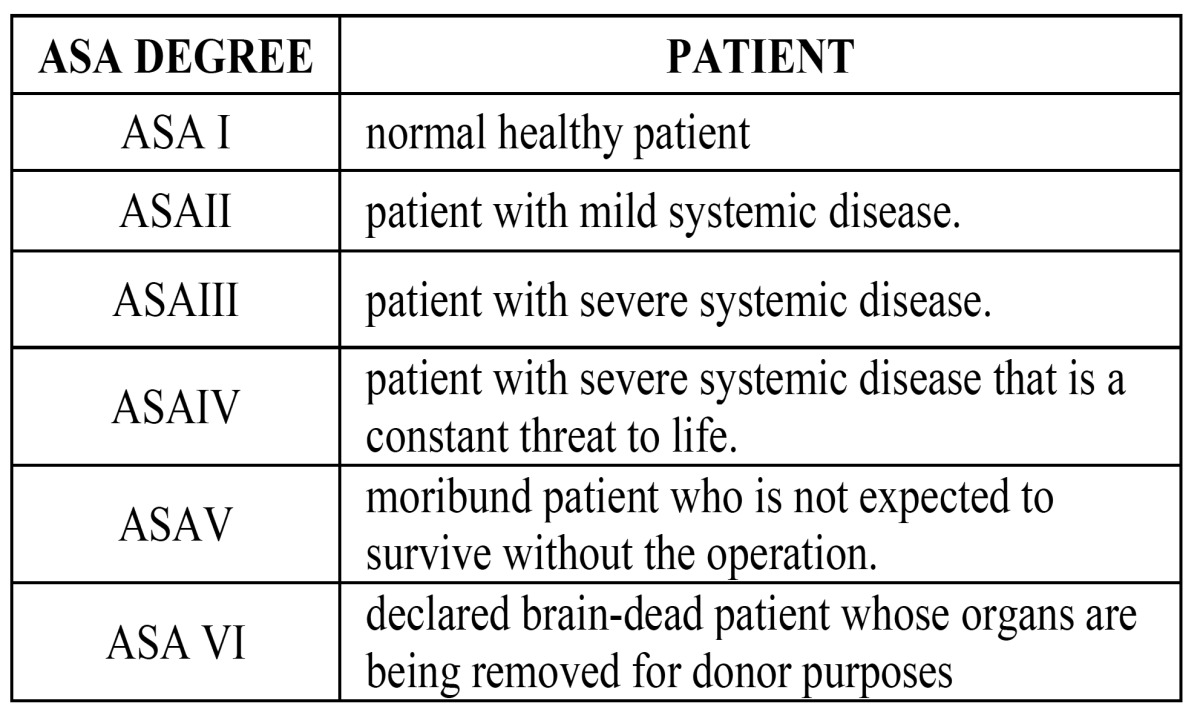
ASA patients classification.

The anaesthesiologist will establish the preoperative protocol with minimum 6 fasting hours and suppression or not, of patient’s underlying medications, depending on patient´s disease and type of drug. On this visit, an informed consent, for the type of anesthetic technique will be performed.

If it´s necessary to administer some treatment to reduce anxiety and fear, at the time of admission before intervention, premedication and administration way( nasal, rectal or sublingual) will be scheduled (20-22).

With all this we make a second patient reassessment where informed consent is signed to carry out the proposed dental treatment. You give to patient´s family, written preoperative and postoperative recommendations to prevent oversights and errors.

In those cases in which correct exploration is impossible and therefore we can not perform a preoperative treatment plan, parents will be informed and we will proceed to evaluate patient needs and establish a treatment plan when patient is slept, forcing us to make changes in predicted treatment, make quick decisions taking into account needs of patient and degree of cooperation in the later maintenance of treatment .

Patient will enter into health service as an ambulatory surgery proceed,a couple of hours before surgery. In these cases treatments are usually performed along morning so patient could remain as shortest as possible in hospital, and they can return sooner to their usual environment.

At the moment of admission at hospital, as a medical order, premedication ordered by anesthesiologist should appear, and it will be administered by nursing staff and, during surgery, certain drugs could be administered parenterally according to patient’s requirements if it ´s needed, as in case of endocarditis prophylaxis in patients with heart disease, analgesics to reduce postoperative pain etc.

After intervention, patient remains with an injecting dropper and under hospital supervision for about four hours and if liquid tolerance is adequate, no vomiting and no complications appear we will proceed to discharge patient with drug regimen to be followed at home depending on each case of dental treatment performed and the patient’s underlying disease.

With this we will try to interfere as little as possible with patient’s general environment and allows that time at hospital is as minimum as possible. Patient should return for a control treatment in the recommended period and in that time, we will proceed to establish the inspection and maintenance protocol to be followed by the patient in order to minimize the emergence of new diseases. On this visit is we will suggest referrals to other services if it is necessary (8).

In conclusion we have described a sequence of performances for outpatient treatment in handicapped patients with different pathologies whether children or adults, both in private practice at dental clinics and in hospitals, in order not to changetheir routine as much as possible and create a favorable environment to face treatment, prescribing sedation or general anesthesia as a last resource in extreme cases only if patient´s pathology, medication or irregular collaboration, require it.
